# Understanding key drivers and barriers to implementation of the WHO recommendations for the case management of childhood pneumonia and possible serious bacterial infection with amoxicillin dispersible tablets (DT) in Bangladesh: a qualitative study

**DOI:** 10.1186/s12913-020-4982-4

**Published:** 2020-02-24

**Authors:** Mahfuzur Rahman, Jaclyn Delarosa, Sharmin Khan Luies, Kazi Robiul Alom, Manjari Quintanar-Solares, Ishrat Jabeen, Tahmeed Ahmed, Elizabeth Abu-Haydar, Haribondhu Sarma

**Affiliations:** 1 International Centre for Diarrhoeal Disease Research, Bangladesh (icddr,b) 68, Shaheed Tajuddin Ahmed Sarani, Dhaka, 1212 Bangladesh; 2 PATH, 2201 Westlake Avenue, Suite 200, Seattle, WA 98121 USA; 3 Department of Anthropology, University of Rajshahi, Rajshahi, Bangladesh; 4 Research School of Population Health, the Australian National University, Acton, ACT 2601 Australia

**Keywords:** Childhood pneumonia, Possible serious bacterial infection, Oral amoxicillin, Dispersible tablets, WHO recommendations, Bangladesh

## Abstract

**Background:**

Pneumonia and possible serious bacterial infection (PSBI) are leading causes of death among under-five children. The World Health Organization (WHO) issued global recommendations for the case management of childhood pneumonia and PSBI when referral is not feasible with oral amoxicillin. However, few governments to date have incorporated child-friendly amoxicillin dispersible tablets (DT) into their national treatment guidelines and policies. We aimed to understand the key drivers to the implementation of WHO recommendations for childhood pneumonia and PSBI using amoxicillin DT in Bangladesh.

**Methods:**

A qualitative study was conducted from October 2017 to March 2018 in two districts of Bangladesh. Interviews were completed with 67 participants consisting of government officials and key stakeholders, international development agencies, health service providers (HSPs), and caregivers of young children diagnosed and treated with amoxicillin for pneumonia or PSBI. Data were analyzed thematically.

**Results:**

Policies and operational planning emerged as paramount to ensuring access to essential medicines for childhood pneumonia and PSBI. Though amoxicillin DT is included for National Newborn Health Programme and Integrated Management of Childhood Illnesses in the Operational Plan of the Directorate General of Health Services, inclusion in Community-Based Healthcare Project and Directorate General of Family Planning policies is imperative to securing national supply, access, and uptake. At the sub-national level, training on the use of amoxicillin DT as a first line intervention is lacking, resulting in inadequate management of childhood pneumonia by HSPs. Advocacy activities are needed to create community-wide demand among key stakeholders, HSPs, and caregivers not yet convinced that amoxicillin DT is the preferred formulation for the management of childhood pneumonia and PSBI.

**Conclusion:**

Challenges in policy and supply at the national level and HSP preparedness at the sub-national levels contribute to the slow adoption of WHO recommendations for amoxicillin DT in Bangladesh. A consultation meeting to disseminate study findings was instrumental in driving the development of recommendations by key stakeholders to address these challenges. A comprehensive and inclusive evidence-based strategy involving all divisions of the Ministry of Health and Family Welfare will be required to achieve national adoption of WHO recommendations and country-wide introduction of amoxicillin DT in Bangladesh.

## Introduction

Globally, lower respiratory tract infections and neonatal sepsis are leading causes of death among children under 5 years old [1]. In Bangladesh, they are the key drivers of mortality, accounting for 15 and 13% of under-five deaths, respectively [2]. Pneumonia continues to be the single most important cause of under-five child mortality, yet only 42% of caregivers of children who have symptoms of pneumonia seek care [3]. Even if treatment is sought, inadequate second-line antibiotics often are used, and more can be done to ensure rational administration of and adherence to medication regimens in Bangladesh [4].

To address these issues, several national interventions for prevention of illness have been implemented in Bangladesh, such as the Global Alliance for Vaccine and Immunization-supported Expanded Programme on Immunization and provision of vaccines starting at birth to prevent targeted infectious diseases, including pneumonia [5, 6]. For management of illness, guidance for inpatient care of severe cases of infection in childhood is available, but this type of care is not often possible because of lack of resources [7], including a shortage of skilled health care workers [8]. Furthermore, patient referral is not always feasible due to inaccessibility, unacceptability, and unaffordability for families [9, 10]. However, outpatient care frequently can be provided, and global clinical and programmatic guidance is available for outpatient management of childhood pneumonia and possible serious bacterial infection (PSBI) in infants [11]. Guidance was provided by the World Health Organization (WHO) in the 2014 revision of the Integrated Management of Childhood Illnesses (IMCI) guidelines for the management of fast breathing and chest in-drawing pneumonia using oral amoxicillin [12], and in the 2015 guidelines for managing PSBI using gentamicin and oral amoxicillin in outpatient settings when referral is not feasible [13].

In 2009, WHO had updated its Model List of Essential Medicines to include the solid oral dispersible tablet (DT) dosage form of amoxicillin (250 mg), and this is now the preferred formulation for outpatient treatment of childhood pneumonia and is being considered for PSBI in young infants. Although there are other pediatric dosage forms of oral amoxicillin, such as drops and dry syrup, dispersible tablets have several advantages over other formulations of amoxicillin. They are cheaper than amoxicillin dry syrup; have logistical and supply-chain advantages in terms of volume and weight; help patients with difficulty in swallowing; do not require refrigeration; and facilitate and simplify management of illness because of greater dosage accuracy compared with the dry syrup, which must be manually measured and properly stored in a cool area [14]. In the treatment of childhood pneumonia, UNICEF reported that shifting the demand from amoxicillin dry syrup to DT has resulted in worldwide cost savings of US$ 8.4 million during 2013–2015 as well as further savings from reduced distribution costs [14].

Through the UN Commission on Life Saving Commodities, significant progress has been made in the dissemination of WHO treatment policies globally and in policy change for childhood pneumonia and PSBI at the national level in target high-burden countries [15]. The accomplishments in these countries include updating lists of essential medicines to include amoxicillin DT, registering products, coordinating procurement and supply-chain logistics, revising clinical treatment guidelines, developing and delivering training for health workers, developing appropriate job-aids and support materials, and monitoring and evaluating policy implementation and impact [15]. However, these changes in policies and practices must be accepted and implemented at the primary healthcare level to achieve impact. Significant policy adoption gaps persist at the national and sub-national levels, and implementation lags at the primary level, preventing widespread access to, uptake of, and appropriate use of amoxicillin DT [16].

In Bangladesh, international development partners and non-governmental organizations have provided technical and financial support toward adoption of the WHO recommendations. For example, the United Nations Children’s Fund (UNICEF) and WHO Bangladesh country office have provided technical support to the Ministry of Health and Family Welfare (MOHFW) to revise the IMCI training manual according to the WHO recommendations. Save the Children has generated evidence on how to manage cases of PSBI at the primary healthcare union sub-center level, and a national comprehensive newborn care package (CNCP) guideline has been developed accordingly. Research by icddr,b in collaboration with PATH (Seattle, USA) assessed feasibility, acceptability, and usage of introducing amoxicillin DT job aids and user-friendly product presentations into the public and private healthcare system in Bangladesh [unpublished observations; Haribondhu Sarma, Emily Gerth-Guyette, Syaket Ahmed Shakil, Kazi Robiul Alom, Elizabeth Abu-Haydar, Methelda D’ Rozario, Md. Tariqujjaman, Shams El Arifeen, Tahmeed Ahmed]. However, despite significant progress and a supportive policy environment for childhood pneumonia and PSBI treatment with oral amoxicillin, barriers to full adoption and implementation persist in Bangladesh and need to be better understood and addressed.

The MOHFW in Bangladesh provides healthcare services for the case management of childhood pneumonia and PSBI in the primary healthcare setting through both the Directorate of Health Services (DGHS) and Directorate of Family Planning (DGFP), under three Operational Plans. Two Plans are managed by DGHS, namely maternal neonatal child and adolescent health (MNC&AH) and community-based healthcare (CBHC), and one Plan is managed by DGFP, namely maternal, child, reproductive and adolescent health (MCR&AH). Our approach in the current study was to examine the status of adoption and implementation of the WHO recommendations in these directorates, as well as at the primary health care level. We employed qualitative approach for the assessment.

The aims of this study were to understand the key drivers for implementation of the WHO recommendations for the case management of childhood pneumonia and PSBI with amoxicillin DT and to generate evidence to strengthen newborn and child health programs in Bangladesh. We anticipate that findings will help policy-makers develop strategies for overcoming challenges to implementation of the WHO recommendations in Bangladesh and other low- and middle-income countries.

## Methods

### Study objectives and design

The primary objectives of this study were to understand the facilitators and barriers to introducing amoxicillin DT for the treatment of childhood pneumonia and PSBI according to WHO recommendations and to understand current management of these childhood illnesses at the primary care level. We conducted a qualitative descriptive study, using interviews, secondary document reviews, and a stakeholder consultation meeting. Data were collected via interviews and document review from October 2017 to March 2018. The consultation was held in July 2018.

### Study sites

The study was conducted in Bangladesh both at the national level and in the districts of Khulna and Lakshmipur, specifically in two sub-districts: Digholia in Khulna and Ramganj in Lakshmipur. The sub-districts were selected based on recent IMCI or CNCP training and distribution of amoxicillin DT. Both sub-districts distributed amoxicillin DT that was donated by UNICEF to public healthcare facilities in 2016. Health service providers (HSPs) in Khulna had received training in 2017 on the revised national IMCI protocol. None of the HSPs in Lakshmipur received such training, although they received training on CNCP guidelines beginning in March 2015.

### Study population

Considering different categories of the respondents we determined that a total of 69 participants from a wide range of perspectives and points of view would be sufficient to interview for the attainment of data saturation for each category. The study population was recruited from three groups: (1) key stakeholders at the national and district levels, who were involved in policy-making, procurement, supply, distribution, and promotion of amoxicillin DT; (2) HSPs at the sub-district level, including Medical Officers, Sub-assistant Community Medical Officers, Community Healthcare Providers, Family Welfare Visitors, private practitioners, drug-sellers, and pharmacists who provide primary healthcare for children with pneumonia or PSBI, and Family Welfare Assistants and Health Assistants who identify and refer PSBI cases at the community level; (Supplementary file 1) and (3) caregivers in the two sub-districts of children under 5 years old who were diagnosed with pneumonia or PSBI in the previous 4 weeks and received any formulation of amoxicillin.

We applied a purposive sampling method to select study participants. At the national and district levels, key stakeholders were identified and selected based on information provided by government officials from DGHS and DGFP during a preliminary meeting with the study team. For HSPs, we tried to maximize the variation in terms of number of years of job experience, while for caregivers, we looked for variation in experience of using different formulations of amoxicillin. A convenience sample of HSPs was recruited from 18 facilities based on accessibility and proximity to the research team. We selected HSPs who were present in the facilities on the day of data collection. We included those who provided care to children below 5 years of age and gave consent to participate in the study. The rural and peri-urban facilities were selected to represent different tiers of the health service delivery system (i.e., sub-district, union, community, and private facilities). Pneumonia and PSBI cases were identified from the registry of the HSPs at the public facilities. HSPs provided the contact information of caregivers of children under 5 years old who were provided treatment for pneumonia or PSBI and received amoxicillin from them in the previous weeks.

### Data collection

Semi-structured discussion guides were developed, pre-tested, and refined before use in interviewing key informants, HSPs, and caregivers of children (Supplementary file 2). We conducted key informant interviews (KII) with national and district level participants and in-depth interviews (IDI) with HSPs at their workplaces. Following initial interviews, six stakeholders at the national level and two stakeholders at the district level were interviewed a second time to clarify the findings. Caregivers of children were interviewed via IDI at the household level. Interviews were audio-recorded and transcribed verbatim on the day of the interview; one respondent declined an audio-record of the interview. Average duration of an interview was 28 min. Three rounds of interviews were conducted, and after each round the study team reviewed emerging themes, discussed the findings, and revisited the interview guides. This process helped to synthesize findings and identify the point of data saturation, at which time that type of participant interview was concluded.

We asked a subset of HSPs who provided treatment for childhood pneumonia from both sub-districts and HSPs who provided treatment for PSBI from Ramganj, Lakshmipur, about their knowledge on diagnosis and provision of treatment by asking specific questions on what signs and symptoms they used to identify PSBI, pneumonia, or severe pneumonia for referral and the proper dosage instructions for medications. The accuracy of their responses was compared to the national standard treatment guidelines for childhood pneumonia and PSBI.

We also reviewed relevant documents identified during the interviews or visits by the research team to health facilities, for understanding current policy and management of childhood pneumonia and PSBI. The relevant documents included reporting forms, registers and revised IMCI guideline. We also searched literature on the operational plan relating to maternal, newborn and child health through internet. From the documents we extracted data on the children with pneumonia treated with different formulations of amoxicillin, supply and distribution of amoxicillin at the facilities, instructions provided to the HSPs on case management of childhood pneumonia and PSBI, and operational plan of MOHFW to addressing childhood pneumonia and PSBI.

### Data management and analysis

A priori themes were defined in advance based on research questions, and additional sub-themes were added as derived from participant responses. Interviewers read the transcripts daily to identify issues for further exploration in the next set of interviews. Three members of the research team coded the transcripts and compared results to check inter-coder reliability and agreement and to resolve discrepancies. We prepared a matrix table in Microsoft Word for data display to identify themes and sub-themes emerging from the interviews (Table 2). Some interviewee comments are presented verbatim to demonstrate complex views and ideas of respondents and to illustrate the themes. Data collected from interviews were triangulated with the findings from the consultation meeting with stakeholders. Data extracted from the documents were also verified during interviews and the consultation meeting.

### Consultation meeting with stakeholders

We disseminated the interview findings to major stakeholders at a consultation meeting in July 2018. The participants included some of the interviewees and some DGHS and DGFP officials who had not been interviewed as well as international development partners, members of professional bodies, and international non-governmental organizations (INGOs). The purpose of the consultation meeting was to understand additional perspectives on the key findings and receive further inputs on the findings. Thus, we presented and verified the findings derived from the interviews. One of the investigators of the study moderated the meeting and facilitated the discussion using a discussion guide. The discussion guide covered the topics on adoption of WHO recommendations in policy, revision of IMCI guideline, procurement, supply and distribution related barriers and facilitators, and future recommended strategies. Stakeholders discussed the challenges and opportunities for implementation of the WHO recommendations that emerged from the study. The consultation meeting generated recommendations to improve national availability and uptake of amoxicillin DT treatment for childhood pneumonia and PSBI. After the consultation meeting, a discussion summary was shared with the stakeholders through email for their further input.

## Results

A total of 67 study participants at the national and district levels were interviewed, at which point no new themes emerged, indicating that data saturation had been reached (Table 1). Twenty-four KIIs were conducted with 16 national stakeholders and 8 district stakeholders. Thirty-one HSPs from a total of 18 facilities were enrolled and interviewed with IDIs. Two pharmacists and two storekeepers were included as interviewees in the HSP group in order to understand their view of the use and administration procedure for amoxicillin DT (Table 1). Eighteen HSPs who provide treatment for childhood pneumonia from both sub-districts, and 9 HSPs who provide treatment for PSBI from Ramganj, Lakshmipur were assessed for their knowledge on diagnosis and provision of treatment. IDIs were conducted with six caregivers of children from each sub-district who had received amoxicillin treatment from HSPs for pneumonia and PSBI. Among these caregivers, three had used amoxicillin DT and two had used the dry syrup in Khulna; in Lakshmipur, two had used DT and four had used drops.

**Table 1:** Stakeholders and health service providers interviewed

Key Stakeholders from the national and sub-national levels		Health Service Providers interviewed from different types of facilities			
National Level	Sub-national level Khulna and Lakshmipur	Digholia, Khulna	Ramganj, Lakshmipur	Facility type [Ownership, location]	Total number of facilities visited
National Newborn Health Program & Integrated Management of Childhood Illness, Directorate General of Health Services (DGHS) [3] Directorate General of Family Planning (DGFP) [1] UNICEF [2] Bangladesh Pediatric Association [2] Save the Children [2] Central Medical Store Depot, DGHS [2] Systems for Improved Access to Pharmaceuticals Services [1] Community Based Healthcare [1] Square Pharmaceuticals [1] WHO Bangladesh [1]	Civil Surgeon [2] Upazila Health & Family Planning Officer [2] Upazila Family Planning Officer [2] District Storekeeper [2]	Medical Officer [2]	Medical Officer [2]	Upazila (sub-district) Health Complex [Public, Rural]	2
		Sub Assistant Community Medical Officer [2]	Sub Assistant Community Medical Officer [1]		
		^a^Upazila Health Complex Pharmacist [1]	^a^Upazila Health Complex Pharmacist [1]		
		^a^Upazila Health Complex Storekeeper [1]	^a^Upazila Health Complex Storekeeper [1]		
		Sub Assistant Community Medical Officer [1]	Sub Assistant Community Medical Officer [2]	Union Sub-Centre [Public, Rural]	4
		Family Welfare Visitor [1]	Family Welfare Visitor [1]		
		Sub Assistant Community Medical Officer [1]	Sub Assistant Community Medical Officer [1]	Health and Family Welfare Centre [Public, Rural]	2
		Community Health Care provider [2]	Community Health Care provider [2]	Community Clinic [Public, Rural]	4
		Health Assistant [2]	–		
		–	Family Welfare Assistant		
		Private Practitioner [2] Drug Seller [2]	Private Practitioner [2]	Private drug shops and chamber of private practitioner [Private, Rural and Urban]	6

^a^The storekeepers and the pharmacists were not directly involved in case management of childhood pneumonia or PSBI. They were providing services at the sub-district level in supply and distribution of amoxicillin

Findings from the interviews revealed opportunities and challenges in achieving full national policy adoption of amoxicillin DT, preparedness of HSPs for case management of childhood pneumonia and PSBI, and ensuring quality of service delivery at primary healthcare facilities. The results also revealed challenges on the demand side, from interviews with caregivers. The themes that emerged from analyzing interviews with the three groups of participants in this study are presented in Table 2 according to the five domains of the consolidated framework for implementation science (CFIR) [17] and discussed below.

**Table 2:** Themes and sub-themes mapped onto 10 CFIR constructs in 5 different CFIR domains for understanding the status of adoption and implementation of WHO recommendations on management of childhood pneumonia and PSBI

Themes	Sub themes	Related domains
National policy adoption of the WHO recommendations for childhood pneumonia and PSBI	Factors that influence decision-making and the policy process	Inner setting: leadership engagement
	Collaboration and support from international development partners	Outer setting: external policy and incentives Outer setting: cosmopolitanism
	National procurement planning and coordination	Outer setting: external policy and incentives Outer setting: cosmopolitanism
Preparedness of health service providers at primary healthcare facilities	Training and barriers to organizing and selecting participants for training	Inner setting: available resources
	Supply and distribution of amoxicillin DT	Inner setting: readiness for implementation Inner setting: implementation climate
	Availability of treatment guidelines or job-aids	Inner setting: available resources
	Availability of basic equipment	Inner setting: available resources
	Case management of childhood pneumonia and PSBI	Characteristics of individuals: knowledge and beliefs about the intervention
	Perception of amoxicillin treatment	Characteristics of individuals: knowledge and beliefs about the intervention Inner setting: Access to knowledge and information
Experience of caregivers with pneumonia and PSBI and treatment modalities	Healthcare-seeking behavior	Implementation process: engaging
	Perceptions and preference for amoxicillin treatment	Intervention characteristics: relative advantage Characteristics of individuals: knowledge and beliefs about the intervention Inner setting: Access to knowledge and information

### National policy adoption of the WHO recommendations

Our review of documents showed that of the three Operational Plans under the two MOHFW directorates, only one—the MNC&AH—has adopted the WHO recommendations on management of childhood pneumonia and PSBI. Interviews and consultation meeting with national level stakeholders revealed that the drivers for adoption included proactive leadership from the national IMCI program (inner setting) and advocacy, technical, and resource support from international development partners (outer setting). On the other hand, lack of coordination in budget planning and development of Operational Plans between the DGHS and DGFP, and between the IMCI program and the Central Medical Stores Depot (which is involved in national procurement of medicines and equipment), were the key challenges to full adoption and implementation of WHO recommendations. UNICEF and WHO played important roles in sensitizing policy-makers and supporting the policy process that led to the MNC&AH adoption. Their important advocacy efforts (outer setting) included presenting global evidence and new WHO guidelines to relevant stakeholders such as members of Bangladesh Pediatric Association, policy-makers, and representatives from INGOs and research organizations. A health manager from UNICEF Bangladesh, who played a key role in the policy adoption process, remarked:

*“It took a long time to sensitize the paediatricians as they had a tendency to provide higher antibiotics and they were not agreeing with the new guideline so it took almost 6 months to convince them. So, UNICEF, along with the Government played crucial role to convince them taking the global evidences and whatever questions that they had you (we) needed to give the answers as well. So you know, here comes the role of UNICEF in setting this up … that kind of technical support we give … ”*

Our document review and interviews with national level stakeholders found that in Bangladesh, two committees are involved in the national protocol or guideline revision process: the National Technical Working Committee (NTWC) and the Core Committee. Representatives from the Bangladesh Pediatric Association, an organization of thousands of pediatricians, have been working through the NTWC with the government of Bangladesh and INGOs, including WHO and UNICEF, on policy issues for child health in Bangladesh. First, all issues regarding treatment of childhood pneumonia and PSBI were discussed in the NTWC, then the IMCI protocol was approved by the Core Committee. Upon understanding the importance of amoxicillin DT and its listing as one of the 13 life-saving commodities of the UN Commission, the DGHS-IMCI authority agreed to include amoxicillin DT in the treatment for childhood pneumonia in their MNC&AH Operational Plan for 2017–2022. The IMCI authority also updated the IMCI training manual in 2016, with technical support from UNICEF, to include signs and symptoms of pneumonia and updated PSBI information according to the WHO recommendations. However, the revised manual did not include specific dosing instructions for amoxicillin DT. Table 3 summarizes the status of adoption and implementation and influential factors.

**Table 3:** Status of adoption and implementation of WHO recommendations for the case management of childhood pneumonia in Bangladesh

Status of compliance with WHO recommendations	Influential factors
Updated national IMCI training manual (2016) following the revised 2014 WHO protocol for the case management of childhood pneumonia	Support from UNICEF, WHO, and other international development partners such as Save the Children.
Budget allocation for amoxicillin DT included in MNC&AH Operational Plan 2017–2022 under DGHS	Proactive leadership of national IMCI program
Delayed national procurement of amoxicillin DT under IMCI program and limited supply to select districts provided by UNICEF for interim period	Lack of procurement planning between Central Medical Stores Depot and IMCI program augmented by temporary support from UNICEF
Budget allocation for amoxicillin DT not included in Operational Plans for MCR&AH under DGFP and CBHC Project under DGHS	Lack of communication and coordination among different operating divisions under MOHFW

The status of adoption and implementation of the WHO recommendations for the case management of PSBI is similar to that for pneumonia, with only the MNC&AH Operational Plan under the DGHS having adopted the policies (Table 4). According to the CNCP protocol based on WHO treatment recommendations, infants with signs and symptoms of PSBI were to be referred to the Upazila (sub-district) Health Complex as the first point of contact. However, under a pilot program in four districts, including Lakshmipur, with the support of Save the Children, the government designated union facilities (i.e., Health and Family Welfare Centres, Union Sub-Centres) as the first points of contact, after CNCP guidelines were implemented. In the pilot areas, the first pre-referral dose of gentamicin by intramuscular injection and amoxicillin paediatric drops are administered for PSBI, and the cases that did not accept referral to the Upazila level were treated with 7 days of amoxicillin pediatric drops, along with 2 days of once-daily intramuscular gentamicin by the Sub-assistant Community Medical Officer.

**Table 4:** Status of adoption and implementation of WHO recommendations for the case management of PSBI in Bangladesh

Status of compliance with WHO recommendations	Influential factors
Health and Family Welfare Centers and Union sub-Centres identified as the service delivery point for outpatient case management of PSBI by the HSPs when referral to a hospital is not feasible	Evidence on case management of PSBI using amoxicillin drops and intramuscular gentamycin generated from the pilot program through partnership with Save the Children
Amoxicillin DT included in OP of MNC&AH under DGHS as an alternative to pediatric drops but is not mentioned in the treatment table of the CNCP training manual	Members of the Bangladesh Pediatric Association and HSPs are unwilling to use amoxicillin DT instead of drops due to lack of operational research

Amoxicillin DT was included in the MNC&AH Operational Plan under DGHS as an alternative to pediatric drops for PSBI but was not listed in the Operational Plans of CBHC under DGHS and MCR&AH under DGFP. In addition, amoxicillin DT was not specified in the treatment table of the CNCP training manual (2014). Through interviews and the consultation meeting, we found that the Bangladesh Pediatric Association and HSPs have not been convinced to use amoxicillin DT instead of drops for the case management of PSBI, both because of lack of clear dosing instructions and scarcity of clean water for dispersal of the tablet. A member of Bangladesh Pediatric Association stated:

*“We need the evidence from WHO and expert opinion to introduce amoxicillin DT for managing PSBI. Before that we need to ensure the proper dose (both schedule and quantity) with the assistance of National Technical Working Committee (NTWC). Also, we need to inform the service provider through training and alert the caregivers for using pure water when dispersing the tablet.”*

### Preparedness of health service providers at primary healthcare facilities

Interviews with HSPs indicated major challenges with their preparedness to manage cases of childhood pneumonia and PSBI at primary healthcare facilities, including inadequate training, lack of basic equipment and guidelines or job-aids, and insufficient supply of medicines (inner setting). Although the training for HSPs is different for the two illnesses, the equipment (i.e., stethoscope, thermometer, weighing scale, and respiratory rate counter) for diagnosis is the same.

#### Training

According to documentation (training participant list) from Upazila Health Complex, in Digholia, Khulna, 113 of 114 HSPs had received training on the revised IMCI protocol for case management of childhood pneumonia. However, only 23 of 167 HSPs in Ramganj, Lakshmipur, received training on CNCP for case management of PSBI at union level facilities when referral to the Upazila level is not feasible, because most of them did not have authority to treat children with PSBI. Another major barrier to organizing the training was staff turnover. A provider from a sub-district level healthcare facility mentioned:

*“Usually a staff member who has received ToT [training of trainers] is responsible for conducting training at the Upazila level but, in case of training on the revised IMCI protocol, I did not receive the ToT. As the actual trainer (who received ToT) has been transferred in somewhere else, they (authority of the facility) had to carry forward the programme.”*

#### Supply and distribution of amoxicillin

To date, amoxicillin DT has not been procured by the MOHFW due to lack of communication, procurement planning, and coordination between the IMCI health services division and Central Medical Stores Depot, the procurement entity for DGHS. Seventy-seven thousand three hundred amoxicillin DTs (one-fourth of the total district supply) were supplied to Digholia, Khulna, and 10,000 amoxicillin DT (one-sixth of the total district supply) were supplied to Ramganj, Lakshmipur with the support of UNICEF. However, the district reserve stores delayed distribution of the tablets because health facilities had amoxicillin dry syrup and capsules available. Interviews revealed there was a lack of clarity from UNICEF and MOHFW on the distribution plan for amoxicillin DT. As a result, DTs were distributed only to the Upazila Health Complex for outpatient care in Khulna. In Lakshmipur, DTs were distributed to the Upazila Health Complexes as well as for use by community-based HSPs in Community Clinics.

#### Availability of treatment guidelines or job-aids

Most of the HSPs providing treatment for childhood pneumonia and PSBI mentioned that they had treatment guidelines or job-aids (inner setting) available for the case management of childhood pneumonia or PSBI. However, only two HSPs at Ramganj, Lakshmipur, demonstrated possession of the 2014 CNCP guideline, and one HSP in Digholia, Khulna, presented the 2017 IMCI manual. One HSP indicated that the manual was not always necessary as he was comfortable enough to diagnose the patients without the manual, stating:

*“We are now accustomed to seeing that (patient)...we can assume their weight and provide the dose accordingly. You see, there are so many patients; all of them do not have time to wait...”*

#### Availability of basic equipment

Out of 18 HSPs in the 18 healthcare facilities visited, 14 had a stethoscope, 11 had a thermometer and weighing scale, and 5 had a respiratory counter (inner setting). Upon asking how an HSP who did not have respiratory counter at a Community Clinic measures the breathing rate, the HSP responded:

*“I cannot count breathing rate accurately, I can understand it normally observing the child (with naked eyes). There is no supply of stop watch (respiratory counter)”*

#### Case management of childhood pneumonia

Only half of HSPs interviewed could state that pneumonia was diagnosed by assessing fast breathing or chest in-drawing and most (13 out of 18 HSPs) could not state the method for diagnosing severe pneumonia for referral (characteristics of individuals). Only one HSP identified the correct oral amoxicillin treatment for pneumonia. Six out of the 18 HSPs perceived amoxicillin as non-effective due to exposure to second- and third-line antibiotics provided by drug sellers and non-formal providers before arrival of the patient at the facilities.

Among the HSPs interviewed in Digholia, Khulna who were involved in providing treatment for childhood pneumonia were MOs, SACMOs and CHCPs. Some of them received training on revised IMCI guideline and one-third of them could not remember if they received the training or not. More than half of the HSPs could not mention the correct dose of amoxicillin for the case management of childhood pneumonia. They mentioned diversified doses of amoxicillin as per child’s age; the frequency they mentioned was thrice a day, which was not consistent with the guideline.

In Ramganj, Laksmipur, one-third of the HSPs interviewed were able to correctly diagnose pneumonia but only 2 HSPs could state the criteria for diagnosis of severe pneumonia for referral. However, none of them could mention the correct dose of amoxicillin in the treatment for childhood pneumonia.

Factors impeding amoxicillin DT use included availability of other antibiotic formulations and lack of instructions and training for HSPs on how to use it. The IMCI training manual includes oral amoxicillin (dry syrup or tablet) but does not mention amoxicillin DT. A Community Healthcare Provider from a Community Clinic, who received training on revised IMCI protocol, stated:

*“Use of amoxicillin DT was not discussed in the training.”*

#### Case management of PSBI

All 9 HSPs noted above who provided case management for PSBI could state the correct diagnosis following the instructions in the CNCP, although only 5 of them were able to state the correct oral amoxicillin treatment (characteristics of individuals). Those who were able to state the correct oral amoxicillin were the SACMOs and MOs and most of them received training on CNCP guideline. All children diagnosed with PSBI by these HSPs were provided amoxicillin pediatric drops, and these HSPs had never used amoxicillin DT for the case management of PSBI and could not compare DT to other amoxicillin formulations.

### Experience of caregivers with pneumonia and PSBI in children and treatment modalities

Among the twelve caregivers of under-five children that were interviewed in both sub-districts, most of them could not state the danger signs of pneumonia (characteristics of individuals). One caregiver correctly mentioned chest in-drawing and fast-breathing as signs of pneumonia. Three caregivers mentioned cough and cold, fever, or suspected infections in the chest as the signs and symptoms of pneumonia. Since PSBI is not a familiar term to the caregivers, we asked the four caregivers whose children suffered from PSBI in Ramganj, Lakshmipur, what signs and symptoms triggered them to seek treatment for their children. Vomiting with difficulty in feeding, breathing problem, and vomiting with breathing problem were signs and symptoms mentioned by caregivers.

#### Healthcare-seeking behavior

We explored health seeking behavior (implementation process) of the caregivers of the children with pneumonia and PSBI. First contacts for half of caregivers were public facilities (either Upazila Health Complex or Community Clinic). However, one-fourth of the caregivers sought care from village doctors as the first contact. Some of caregivers first sought care from pharmacies and private practitioners. One caregiver treated her child with home remedies before visiting a public facility. In Digholia, most caregivers mentioned that they did not provide any home remedies but took the child directly to the health facilities (i.e., government hospital or Community Clinic). In Ramganj, a major portion of the caregivers mentioned that they did not provide any home remedies but took the child first to the village doctors or private practitioners or called the provider to visit the child in their residence. One of the caregivers took her child first to the pharmacy/drug shop, and another took her child to the district hospital.

#### Perceptions and preference for amoxicillin treatment

Some caregivers interviewed reported that they received instructions from HSPs on how to dissolve the DT in water (inner setting). Among 5 amoxicillin DT users interviewed, one preferred DT (intervention characteristics) but four of them mentioned that they did not find it easy to administer. The caregiver who preferred DT mentioned that she liked it because her child, who was 6 or 7 years old, could easily swallow it, although she did not receive any instruction on how to administer it. Four caregivers mentioned that they preferred dry syrup over DT as they perceived that it was difficult to feed DT to their children because of its bitter taste (characteristics of individuals). Two of them added that it was difficult to measure the dose of DT compared to dry syrup, because the HSPs instructed them to break the tablets into two or three pieces.

Among the four amoxicillin paediatric drop-users, two had not heard about amoxicillin DT. The other two perceived that their children would prefer drops over DT. One of them added that in order to feed drops, she would have to prepare it once, but for DT, she would have to prepare it every time she would feed her child. Two of the 3 caregivers who used amoxicillin dry syrup did not have any preference on the formulation of amoxicillin. A caregiver of a 13-month-old child from Digholia, Khulna said:

*“We give medicine to our children for the improvement of their condition based on the instructions provided by the health service provider; so, it doesn’t matter what the formulation of the drug is,”*

## Discussion

Our study found that the MOHFW in Bangladesh has not fully adopted and implemented WHO recommendations for the case management of childhood pneumonia and PSBI: amoxicillin DT has been included in the MNC&AH Operational Plan under DGHS, but not for the other DGFP and CBHC Project divisions. The national IMCI protocol was revised in 2017 and pneumonia was reclassified based on WHO recommendations; these actions also indicate some progress towards policy adoption. Progress was influenced by international development partners, with UNICEF helping to revise the IMCI training manual and supporting initial availability of the commodity as well as demand creation.

Incomplete policy adoption can be attributed to several barriers, including insufficient coordination among divisions of the MOHFW; lack of central procurement of amoxicillin DT; and perceptions of the efficacy of antibiotics and formulations at the national and district levels. A study suggests that policy decision on evidence-based medicine and clinical practice guideline should be made systematically and participation of different stakeholders including HSPs and even the representatives or advocates of the patients needs to be ensured [18]. Coordination among the relevant stakeholders is instrumental to policy adoption and implementation [19] From information gathered through interviews and a consultation meeting, we have identified several facilitators and opportunities for achieving full adoption of amoxicillin DT for these childhood illnesses at the national level. First, international development partners can play a pivotal role in promoting coordination among the MOHFW and between implementing agencies. Although there are evidences that international development partners are contributing to strengthening health system through implementation research [20] these are in limited scale and their implementation support needs to be expanded.

Next, stakeholders at the national level still have concerns about the suitability of amoxicillin DT administration to the newborns and inconsistent access to clean drinking water necessary for the administration of DT. Therefore, advocacy based on global and local evidence on the suitability of amoxicillin DT for administration to newborns is required to build consensus among stakeholders.

At the sub-district level, we found low preparedness of HSPs to diagnose and treat pneumonia and PSBI, including using amoxicillin DT, demonstrating that inclusion of drugs in policies is not sufficient unless the perceptions of HSPs are changed. Although preferences for other formulations of amoxicillin over DT are noticeable among the HSPs, this also may have resulted from a lack of instructions on how to use it.

HSPs at community levels can play a vital role in integrated management of childhood illness [21, 22], and investments in HSP training, health systems support, and community level activities can result in improvements in the quality of care in health facilities, increase in use of facilities, and higher proportion of sick children taken to an appropriate provider as has been demonstrated by a cluster-randomised study in Bangladesh [23]. Although the country has healthcare facility infrastructure at the community level, training and refresher trainings are lacking for HSPs in primary healthcare settings on understanding signs and symptoms of disease, treatment dosing, and follow-up. These findings are similar to results of a multi-country assessment [15]. We found that because of insufficient training, HSPs were not able to state the correct dose of amoxicillin for the case management of childhood pneumonia in Digholia, Khulna. HSPs in Ramganj, Lakshmipur, were better trained and knowledgeable compared to their counterparts in Digholia, Khulna, likely because of the Saving Newborn Lives training intervention of the government and Save the Children.

In the context of Bangladesh, key challenges to implementing WHO recommendations are rooted not only in policy adoption but also in readiness of the health system to execute the policy; for example, a change in the treatment regimen requires a consistent supply of related drugs to make the change effective. Our study revealed an inconsistent distribution plan for amoxicillin DT that resulted in the supply of amoxicillin DT to include a lower level (Union Sub-centres) in one sub-district, but only to a higher level (Upazila Health Complexes) in another sub-district.

Inadequate supply of medicines also influences the prescription practices of HSPs. It is evident that lack of supplies and medicines in primary healthcare facilities and lack of understanding of the distinct roles of stakeholders can hinder implementation of IMCI guidelines [21, 24]. Inclusion of amoxicillin DT in the essential medicine list of the MOHFW could help ensure supply at all primary healthcare facilities.

### Recommendations

In order to generate insights on how to facilitate full adoption and implementation of the WHO recommendations for the case management of childhood pneumonia using amoxicillin DT, we presented results of our interviews and document review at a consultation meeting of stakeholders. These officials and partners developed several actionable recommendations, listed below.

1. Since acceptability of amoxicillin DT is still a challenge for the case management of PSBI, operational research is needed to generate evidence on the use of amoxicillin DT for infants 0–59 days of age compared to oral drops. Integration of campaign information and education materials on pneumonia and PSBI to increase care-seeking by caregivers is also essential as part of an intervention in operations research.
2. Advocacy initiatives should be undertaken to promote revisions to the MCR&AH Operational Plan under the DGFP and the CBHC Operational Plan under the DGHS.
3. Training and instructions should be provided for HSPs on amoxicillin DT efficacy and use.

## Limitations of the study

This study was carried out in two-sub districts under two districts, thus, the findings generated may not be representative of other districts of the country where the health service providers are managing childhood pneumonia and PSBI following the revised protocol of IMCI. No real-time observations of service delivery were done to understand the practices of health service providers in the facilities. Additionally, the findings from the interviews on case management of childhood pneumonia and PSBI might be subject to recall bias.

## Conclusion

While Bangladesh has made significant progress to reduce preventable newborn and child deaths, key challenges remain at the national and sub-national levels, contributing to slow adoption of the WHO recommendations for the case management of childhood pneumonia and PSBI using amoxicillin DT. These include strengthening coordination for policy adoption, procurement planning, distribution, and use of amoxicillin DT, as well as preparedness of HSPs at the sub-national level to provide health services and manage cases of childhood pneumonia and PSBI. Recommendations to address these challenges were generated from a consultation meeting with key stakeholders. Operations research was requested by stakeholders in order to accelerate adoption and implementation of the WHO recommendations.

## Supplementary information

**Additional file 1.** Supplementary file 1: Qualification of the health service providers.

**Additional file 2.** Supplementary file 2: Semi-structured discussion guidelines.

## Data Availability

The dataset is available with MR, the Principal Investigator of the study in Bangladesh. Non-identifiable data can be accessible upon request subject to approval of the Research Administration Department of icddr,b.

## Abbreviations

CBHC: Community-based healthcare
CNCP: Comprehensive newborn care package
DGFP: Directorate General of Family Planning
DGHS: Directorate General of Health Services
DT: Dispersible Tablet
HSPs: Health service providers
IDI: In-depth interviews
IMCI: Integrated Management of Childhood Illnesses
INGOs: International non-governmental organizations
KII: Key informant interviews
MCR&AH: Maternal, child, reproductive and adolescent health
MNC&AH: Maternal neonatal child and adolescent health
MOHFW: Ministry of Health and Family Welfare
NTWC: National Technical Working Committee
PSBI: Possible serious bacterial infection
UNICEF: United Nations Children’s Fund
WHO: World Health Organization
