# Supplementary material for: Understanding key drivers and barriers to implementation of the WHO recommendations for the case management of childhood pneumonia and possible serious bacterial infection with amoxicillin dispersible tablets (DT) in Bangladesh: a qualitative study
Source: BMC Health Serv Res. 2020 Feb 24;20:142. doi: 10.1186/s12913-020-4982-4 (PMC7041088; doi:10.1186/s12913-020-4982-4)
Supplement: Supplementary file 1 — Additional file 1. Supplementary file 1: Qualification of the health service providers. [file 12913_2020_4982_MOESM1_ESM.doc]

Supplementary File 1: Qualification of the health service providers

| **Designation** | **Qualification** |
| --- | --- |
| Community Health Care Provider | Completed at least 10 years of schooling and received 12 weeks basic training |
| Drug-sellers | Completed 3 months pharmacist course from Bangladesh Pharmacy Council |
| Family Welfare Assistant | Completed at least 10 years of schooling and received basic training of 2 months |
| Family Welfare Visitor | Completed at least 10 years of schooling and 18 months training course |
| Health Assistant | Completed at least 12 years of schooling and received basic training of 1 month |
| Medical Officer | Completed at least Bachelor Medicine, Bachelor of Surgery (MBBS) |
| Pharmacist | Completed at least 10 years of schooling and paramedic course |
| Private Practitioners | Completed at least Bachelor of Medicine, Bachelor of Surgery (MBBS)/ Informal health service providers practicing allopathic medicine (Village Doctor) |
| Sub-assistant Community Medical Officer | Completed at least 10 years of schooling and 3 years diploma course the medical assistant training school |
