# Supplementary material for: Understanding key drivers and barriers to implementation of the WHO recommendations for the case management of childhood pneumonia and possible serious bacterial infection with amoxicillin dispersible tablets (DT) in Bangladesh: a qualitative study
Source: BMC Health Serv Res. 2020 Feb 24;20:142. doi: 10.1186/s12913-020-4982-4 (PMC7041088; doi:10.1186/s12913-020-4982-4)
Supplement: Supplementary file 2 — Additional file 2. Supplementary file 2: Semi-structured discussion guidelines. [file 12913_2020_4982_MOESM2_ESM.doc]

# Guideline for Key Informant Interview with Stakeholders

| Date (DD/MM/YYYY): | Respondent ID number: |
| --- | --- |
| Name of researcher conducting the interview: | |
| Respondent Position/Title: _______________________  Number of years in current position: ______________  Number of years of experience working in health: _______________  Highest level of Education: __________________________ | |
| Stakeholder Group:   | **National:**   - MoH&FW representative:__________ - IMCI - Save the children - MSH - BPA - UNICEF - WHO - CC authorities - Others (specify)__________ | **Sub-National:**   - Civil Surgeon (CS) - Family Planning Officer - Central Store keeper - Medical officer (MO) from CC - Others (specify)____________ | | --- | --- | | |
| Please describe the scope of your role within the health system. Please describe if and how your current position involves the provision of amoxicillin for childhood pneumonia/POSSIBLE SERIOUS BACTERIAL INFECTION . | |
| **Questions** | |
| **Current Health Policies and Practices** | |
| 1. Please describe the organogram of our health system existing across the country. 2. In this organogram which of these facilities/layer provide outpatient care for childhood pneumonia/possible serious bacterial infection using Integrated Management for Childhood Illnesses (IMCI/CNCP)? ` | |
| 1. To gauge awareness of burden of disease, for all sectors:   What are the main causes of death in children under 5 years of age in our country?   1. To gauge awareness of burden of disease, for all sectors:   How big of a problem is childhood pneumonia/possible serious bacterial infection in our country?   1. How does the surveillance for childhood pneumonia/possible serious bacterial infection deaths happen in the country? 2. Is there any special programs that currently focus on child health, and specifically childhood pneumonia/possible serious bacterial infection? If yes, please describe. [Ask if they can share documents.] 3. What is the usual process through which a specific disease or health program get prioritized and adopted in the country? | |
| 1. *To be able to compare with national guidelines:*   What are the health policies that are being followed currently in the country for childhood pneumonia/possible serious bacterial infection diagnosis and treatment? [Ask if they can share documents.]   1. Which type of health workers are trained to diagnose pneumonia/possible serious bacterial infection? 2. Which type of health workers are allowed to prescribe the medication for pneumonia /possible serious bacterial infection? 3. What drug is generally prescribed for outpatient treatment of childhood pneumonia/possible serious bacterial infection?   *Probe: Is amoxicillin routinely prescribed for treatment of pneumonia/possible serious bacterial infection? What other medications are prescribed?*   1. What key challenges exist in making pneumonia/possible serious bacterial infection treatment available and accessible for children in your country? | |
| 1. How often does training on IMCI and childhood pneumonia/possible serious bacterial infection diagnosis and treatment take place for health workers? 2. Were these training provided on the previous or revised IMCI guidelines? [IMCI]   Probe: Who spent this letter to conduct the training? Who was the training intended for? What were the topics covered during the training (was amox DT mentioned)? Who facilitated them? Where there any third partied in charge of these training (esp. Care/UNCIEF)   1. Who is responsible for conducting the training? 2. How does training usually trickle down to the lower level facility health workers? 3. When was the last IMCI training or training on possible serious bacterial infection provided in your area? 4. What educational materials, guidelines or job aids are available for facility-based health workers to diagnose and treat pneumonia/possible serious bacterial infection? [Ask if they can share documents.] 5. If there is an update in guidelines for management of childhood diseases, how do health workers usually learn about them? 6. Can you please mention the availability of resources for correctly diagnosis and management of childhood pneumonia and possible serious bacterial infection?   *Probe: To manage cases correctly, healthcare providers need to have- weight scale, thermometer, watch or respiratory counter, amoxicillin, gentamicin, syringe, needle and disposal of sharp objects. Are these typically available? Can you provide or suggest and documentation of it. What are the procurement cycles like for these equipment (Probe on each of the items except amoxicillin DT)* | |
| 1. Is there planned supervision of facilities on IMCI and pneumonia/possible serious bacterial infection diagnosis and treatment? 2. How often does it happen? 3. When was the last supervision conducted, and what were the findings? 4. What Checklist/tools are used for conducting the supervision? Can you please provide the checklist. | |
| 1. Please describe how caregivers usually obtain the prescribed treatment for pneumonia/possible serious bacterial infection.   *Probe: Given free of charge at the facility? Purchase it elsewhere?*   1. Are caregivers given any printed instructions or written information on how to give the pneumonia/POSSIBLE SERIOUS BACTERIAL INFECTION treatment to their child? | |
| Recently MOHFW has adopted revised WHO guideline for treatment of Childhood pneumonia with amoxicillin DT especially at primary care level. / Recently a pilot program is going on some facilities on PSBI treatment of young infant with gentamicin and amoxicillin in case of refused referral.   1. Could you please inform us the present status of these policy adoption/pilot programs?   *Probe: Change in the OP, change in the guideline, training to follow new treatment regimen.*   1. What has been so far done to adopt this policies/scale up this pilot program for whole country? | |
| **Only for POSSIBLE SERIOUS BACTERIAL INFECTION** | |
| 1. If in PSBI treatment amoxicillin DT instead of amoxicillin syrup/other formulation is wanted to be introduced, in your opinion what kind of changes are required to be done in existing system of practice?   ***Probe:***  *Which professional bodies and stakeholders are needed to be agreed?*  *In policy level what kind of changes are required and who are the concern authorities in MOHFW to do that?*  *What could be the possible steps of advocacy with relevant stakeholders to introduce amoxicillin DT in PSBI treatment?*  *What kinds of evidences are required to show to make agree the key stakeholders about using Amox DT in POSSIBLE SERIOUS BACTERIAL INFECTION treatment?* | |
| **Program and Budget Priorities [PRIVATE SECTOR LEADERS SKIP THIS SECTION] for amoxicillin DT** | |
| 1. Is there any budget allocated for amoxicillin DT in this year? 2. How are decisions made on what items get prioritized in the budget? And how was the budget for amoxicillin DT decided? 3. How does the MOH help coordinate and/or finance childhood pneumonia treatment at the country level? 4. What is the amount of the budget that has already been spent on amoxicillin DT as of today? [Ask if they can share documents.] | |
| **Procurement And Distribution Of Amoxicillin DT** | |
| 1. What initiated/cused the distribution of amoxicillin DT by UNICEF? [UNICEF]   Probe: Who made the decision? Why did UNICEF step in?   1. How were the districts selected and prioritized for distribution of amoxicillin DT? [UNICEF] 2. Please describe the procedure for procuring amoxicillin DT/syrup (quantification and diagnoses considered, tender, purchase, etc.). 3. What is the schedule for procuring amoxicillin DT/syrup? How often are orders placed? 4. What are the main challenges in procuring the amount of amoxicillin DT/syrup needed to cover the country’s needs? 5. When was the last order for amoxicillin DT/syrup placed? [Ask if they can share documents.] 6. Which companies or entities does the country procure the amoxicillin from (syrup in the past, DT currently)? 7. How is it determined which company or entity the amoxicillin is bought from? 8. What are the criteria or condition for distributing amoxicillin DT from Sub-district to Community level? [Central Store Keeper]   Probe: Does the distribution of amoxicillin DT to the UHC/CCs on its availability only?   1. What is your understanding of the advantages and disadvantages of procuring amoxicillin DT instead of syrup? | |
| 1. Who decides what amount of amoxicillin DT/syrup is distributed to each level of facility? 2. Please describe how this decision-making process takes place? What factors are considered to allocate the amoxicillin DT/syrup? 3. Please describe the process through which amoxicillin DT/syrup is physically distributed to health facilities (and pharmacies), particularly the primary care level facilities. 4. Do health facilities ever run out of amoxicillin DT/syrup? What happens then? 5. What is the alternative medicine in case where there is no amoxicillin DT/syrup? 6. In your opinion, how functional are the procurement and distribution processes in the country? What is working and what could be improved? | |
| **Distribution of UNICEF-provided Amoxicillin DT [ONLY for Pneumonia]** | |
| 1. How many packages of amoxicillin DT have been received from UNICEF? 2. How many packages of UNICEF amoxicillin DT were distributed to each facility? Could you please share the distribution list and schedule? 3. Please describe the process through which it was decided the amount of UNICEF provided amoxicillin DT that each facility received (tertiary, secondary, primary levels). 4. Were there any challenges in distribution/receipt of the UNICEF amoxicillin DT at the facility level? 5. What data is collected from facilities on current stock of amoxicillin DT? *Probe : Is the diagnosis for which it was prescribed included in the information collected?* How often is it collected? | |
| **Challenges and Opportunities** | |
| 1. What recommendations do you have for addressing challenges to childhood pneumonia treatment using amoxicillin DT? 2. Are there challenges specific to DT in providing consistent access to pneumonia treatment? Which is the greatest challenge? 3. What would it take to increase the procurement and use of amoxicillin DT in the health system? What policies, resources, or financing would be required to improve access? 4. Is there anything else that you would like to tell me that I didn’t ask? 5. Do you have any questions or comments? | |
| **Document for review** | |
| Supervision checklist, budget, training manual, district-wise DT distribution list | |

Thank the respondent for their assistance.

# Guideline for In-Depth Interview with Health Service Providers (HSP)

| Date (DD/MM/YYYY): | Participant ID number: |
| --- | --- |
| Name of researcher conducting the interview: | |
| Facility Name: | District & Upazila: |
| HSP job title (select one):   - Residential Medical Officer - MO - SACMO: DGFP/DGHS - Pharmacist - CHCP - IMCI corner (type): _____________ - Private Practitioner (type):_________________ - Drug Sellers - Other:______________________________ | Age of the participant:  Number of years in current position:  ______________  Number of years of experience working in health:  _______________  Level of Education:  ___________________  Catchment area:  _____________________ |

| **Question** | |
| --- | --- |
| **Training** | |
| 1. How do you treat children suffering from pneumonia/possible serious bacterial infection? [SACMO, CHCP, etc.] 2. Have you received training on IMCI/CNCP?  - Yes - No  1. When was the last time you received IMCI/CNCP training?  - In the last year - More than a year ago - Never  1. When was the last time you received training on diagnosing and treating pneumonia/possible serious bacterial infection in children?  - In the last year - More than a year ago - Never  1. Who provided that training?  - National IMCI officers - Sub-district officers/Upazila officers - NGO representatives : ______________ - Other : ________________  1. What did you learn in that training about how to diagnose pneumonia/possible serious bacterial infection in children? 2. Did they include in that training how to use amoxicillin to treat pneumonia/possible serious bacterial infection in children?  - Yes - No   If yes, what did you learn about using amoxicillin syrup or dispersible tablets (DT)?   1. Do you feel the training was adequate?  - Yes - No - Other : __________________  1. What do you do when you have questions about pneumonia/possible serious bacterial infection diagnosis and treatment? 2. Do you have a referral system?  - Yes - No - Other : __________________   If yes, when do you refer and why? | |
| 1. If treatment recommendations for any childhood illness changed, how would you find out? 2. What challenges do you face when recommendations change? | |
| 1. On an average how many children with pneumonia/possible serious bacterial infection do you see every month? 2. From where they do come referred from- other facility or to you as first contact? 3. How common do you feel this disease is in your area? 4. How do you manage a child 2 months to 5 years old suffering from childhood pneumonia? 5. How do you manage a child 0 to 59 day’s old suffering from possible serious bacterial infection? 6. In case of pneumonia (non-severe)/possible serious bacterial infection, what treatment do you give? 7. How do you determine the dose?  | - Pneumonia: - Amoxicillin DT:   2-12 months :  13-59 months: | Possible serious bacterial infection:  0-28 days:  Gentamicin  Amoxicillin  29-59 days:  Gentamicin  Amoxicillin | | --- | --- |  - - 1. Others :__________  1. What other drugs do you prescribe to treat pneumonia/possible serious bacterial infection?   (If answer different from guideline/recommended treatment, then ask) What is the reason you prescribe [name of the drug]?   1. How did you learn to prescribe this drug? 2. Do you use any equipment to diagnose and treat childhood pneumonia/possible serious bacterial infection?  - Yes - No - Other: __________________  1. Which equipments do you use for this? (Answer options should include weight scale, watch or respiratory counter, thermometer). | |
| **Supervision and Motivation** | |
| 1. When was the last time that you received any type of supervision?  - In the last year - More than a year ago - Never - Other :   From whom?   - National officer - District officer - Other : ___________________________  1. Did that supervision include asking you questions about the content of the IMCI/CNCP chart booklet?  - Yes - No  1. Did that supervision include asking you questions about pneumonia/possible serious bacterial infection diagnosis and treatment?  - Yes - No | |
| **Knowledge And Practice Of Diagnosis And Management Of Childhood Pneumonia** | |
| 1. How do you know if a child has pneumonia/possible serious bacterial infection? Please name the signs that you would see and hear. 2. What guidelines or other materials do you use to help you in diagnosing pneumonia/possible serious bacterial infection and knowing the treatment the child needs? | *Mark if the HW mentions the following pneumonia signs:*   - Fast breathing _______   [2-12 mo: 50 breaths per min or more;  12 mo – 5 yr: 40 breaths per min or more]  *Mark if the HW mentions the following possible serious bacterial infection danger signs:*   - Difficulty in feeding _______ - Vomits everything _______ - Convulsions _______ - Lethargic or unconscious _______ |
| 1. What treatment would you provide to this child (a child with pneumonia- fast breathing, non-severe) who is 33 months old?   Could you please tell us how you do provide treatment to a 3 kg baby with clinical severe infection signs when the family does not accept referral?   1. Please pretend that I am the child’s caregiver and let me know what you would tell that caregiver.  - Do you usually demonstrate how to give the medicine to the child?   Yes  No  Are there written/printed instructions available for caregivers to take home?  Yes  No | *Mark HW response.*  *Ideally they use the IMCI/ CNCP chart booklet to find answer. Depending on version and on whether they said they received training on use of amoxicillin for pneumonia, treatment should be:*   - 250mg amoxicillin DT 1 tablet/ twice a day/ five days - 250 mg amoxicillin DT 2 tablets/ twice a day/ five days   For possible serious bacterial infection :   - encourage referral to Upazila Health complex - washed hands before injection administration - calculate gentamicin dosage (should be 0.45ml of the 40mg/ml presentation) - prepare the syringe (selected an insulin syringe) - cleaned injection site with antiseptic - injected "vertically" in anterolateral part of thigh [should explain that either thigh can be used, but opposite side when applying vaccines] - dispose correctly of sharps - throw away gentamicin 2ml opened ampoule - reconstitute the amoxicillin drops (adds two teaspoons (10ml) of cooled, boiled water and shakes gently) - calculate amoxicillin dosage (dose should be 1.6ml every 12 hours) - administer the amoxicillin dosage using dropper (calculates that two droppers are needed for each dose: 1.25ml plus 0.35ml) - mark dropper for caregiver - provide TWO bottles of amoxicillin - give the caregiver correct instruction of dosage for home administration of amoxicillin including shaking the bottle before use and appropriate storage (cool place protected from light) - counsel the caregiver (both on how to administer amoxicillin using the markings on the dropper every 12 hours and danger signs)   *Mark HW response, which should include:*   - Explaining the reason for providing the drug - Explaining how to prepare the amoxicillin - Demonstrating how to measure a dose - Asking caregiver to give first dose to child - Instructing that course/dose of the medicine should be completed even if the child feels well - Giving written/printed instructions - Instructing for follow-up.   *Why or why not :*  *If yes, describe instructions the HW hands to caregivers:* |
| 1. Do you think caregivers give their children all of the medication?  - Yes - No  1. What are some of the reasons why they might not complete the treatment? Why or why not. 2. Do caregivers usually return for follow-up visits if they are asked to?  - Yes - No | |
| **For Pneumonia only:**   1. How do you identify whether the child is suffering from severe or mild pneumonia?   Probe: What is the child’s age range for childhood pneumonia? Is this age range same for both severe and mild pneumonia cases? Are they treated differently? How? [SACMO, CHCP, etc.]   1. When a child is diagnosed as mild pneumonia patient, what do you do?  - Provide treatment (if so, why?) - Refer to higher facility.  1. If you provide treatment, which medication do you prefer? Why? 2. How do you know about this treatment and medication? | |
| **Current Use Of Amoxicillin** | |
| 1. For what kind of diseases in children up to 5 years of age do you prescribe amoxicillin?   *(Probe diseases: ear infection, acute malnutrition, red umbilicus in newborn, skin pustules in newborn, other. Probe age groups.)* | |
| 1. What influences your decision on the type of drug you use for pneumonia/possible serious bacterial infection? *(Probe: drug company visits, what pediatricians prescribe in private practice, etc.)* 2. Where do you receive information on pneumonia/possible serious bacterial infection treatment for children? Do you ever receive information from drug companies? What do they tell you? 3. Do you ever follow the recommendations that drug companies give you? | |
| **Perception of Amoxicillin** | |
| 1. What do you think of amoxicillin syrup as a treatment for childhood pneumonia/possible serious bacterial infection?   *(Probe: Is it effective? In your experience how often does it cause side effects? How easy is it for caregivers to use?)*   1. What do you think of amoxicillin DT/Amoxicillin as a treatment for childhood pneumonia/possible serious bacterial infection?   *(Probe: Is it effective? In your experience how often does it cause side effects? How easy is it for caregivers to use?*   1. Is there any difference between these antibiotics (between difference versions of amoxicillin)? Is there any difference between amoxicillin DT with other antibiotics (compared to broad spectrum antibiotics)? [SACMO, CHCP, etc.]   Probe: try to explore other aspects to understand their perception better. Is there any difference between the antibiotics? What are they? Is the concentration, dose administration, duration same or different? If the drugs are provided based on their stock availability, which drug is prescribed the most? Is its stock always available?   1. Have there been occasions when you had amoxicillin DT/amoxicillin available to treat pneumonia (2-59months)/possible serious bacterial infection (0-59days) but decided to give a different medicine? Why? 2. Which presentation of amoxicillin do you prefer for children, syrup or DT?  - Amoxicillin syrup - Amoxicillin DT  1. What or who do you think has influenced how you feel and think about amoxicillin syrup? And DT? | |
| **Supply and Demand Of Amoxicillin DT** | |
| 1. Do you currently have amoxicillin available in the facility? (ask to see document, the type and amount of amoxicillin) 2. Do you always have enough stock for your daily prescriptions? 3. For which other illnesses do you prescribe amoxicillin DT? 4. How do you place your order for amoxicillin DT? 5. When it was last distributed? 6. Do you ever run out of supply for amoxicillin DT? 7. How frequently do you experience a stock out of amoxicillin? 8. Why do you think these (positive or negative outcome) occurs? 9. (If runs out of supply), what did you do then? 10. What happens when you have amoxicillin DT leftover? 11. Why do you think these occur? 12. What needs to improve? | |

| **Monitoring of Amoxicillin Products** |
| --- |
| 1. For HW or pharmacist, as appropriate in the facility visited:   How does this facility monitor and manage the stock of amoxicillin products?  Could you please describe the process, order forms used, who is involved at facility levels, etc.? |
| **Suggestion and Recommendation** |
| 1. What do you suggest for increasing the use of amoxicillin DT for childhood pneumonia and severe infection/possible serious bacterial infection? |
| 1. Is there anything else that you would like to tell me that I may have forgotten to ask? |
| **Documents to Ask for Review** |
| 1. Ask for job aid, brochure, any instruction or guideline that the healthcare providers follow to use amoxicillin DT for the treatment of childhood pneumonia and possible serious bacterial infection, which is available to him during the day of interview. 2. Ask for documents showing the current stock of amoxicillin DT and other formulations.    1. How much received? When?    2. Last month how fast was the stock depleted? |

**Thank the respondent for his/her assistance.**

**Guideline for In-Depth Interview with Caregivers**

| Date: (DD/MM/YYYY) | Participant ID number: |
| --- | --- |
| Formulation of amoxicillin received in immediate past 4 weeks: | Name of researcher conducting the interview: |
| Type of Disease:  Date diagnosis was made (DD/MM/YYYY):  State of recovery: | Relationship of the caregiver to the child:  Age of caregiver:  # of children in the household:  # of siblings the child has:  Age of the child:  Occupation of the caregiver:  # of household members:  Monthly household income: |
| Years of formal education completed:  ☐≤5 years  ☐5-12 years  ☐≥12 years  ☐Other (please specify) __________________ | |

| **Care-seeking practices** |
| --- |
| 1. When your child got sick what was the first thing you did? 2. Did you take your child for care? Where (name of healthcare provider and facility)? Did you seek anyone else for care? 3. How did you know when to take your child to see a healthcare provider/to a health facility? 4. Did you have any challenges getting your child to the healthcare facility/provider? *Probe: distance, cost, healthcare provider attitude, etc* |
| 1. In general, are you satisfied with the care provided by healthcare providers for your child? Why or why not? |
| **Instructions received and perception of their ease of use** |
| 1. What did the healthcare provider do to treat your child? 2. Did the healthcare provider give any instructions? 3. What instructions were you given? 4. Did the healthcare provider ask you to come for follow up? 5. Did the healthcare provider refer you to another health facility? 6. Did you follow the given instructions? What did you do? 7. What happened when you followed them? 8. Did you go back to the healthcare provider? Why or Why not? 9. *If caregiver went to another healthcare provider*, why did you go to this other healthcare provider? 10. Your child recently was diagnosed with pneumonia or possible serious bacterial infection, were you provided treatment with amoxicillin (syrup or DT). 11. Were you provided any formal written instruction with it? 12. *(Take a picture and describe whether they include or not the following: pictorials, dose of amoxicillin, instructions on how to prepare it, number of days to complete, and danger signs, when to go back.)*How confident did you feel in your ability to administer amoxicillin (syrup or DT)? 13. Did you receive a demonstration on how to give amoxicillin (syrup or DT) to your child at home? 14. What would have made it easier for you to follow the instructions? 15. What dosage was prescribed for your child? [Caregiver]  | Pneumonia:  Amoxicillin DT:  2-12 months :  13-59 months: | possible serious bacterial infection:  0-28 days:  Gentamicin  Amoxicillin  29-59 days:  Gentamicin  Amoxicillin | | --- | --- |   Others :__________ |
| **Adherence** |
| 1. During how many days did you give amoxicillin (syrup or DT) to your child? 2. *If 5(pneumonia)/7(possible serious bacterial infection) days not completed*: could you share with me the reasons for giving amoxicillin *x* days instead of 5(pneumonia)/7(possible serious bacterial infection) days? 3. What do you do if the child feels better before finishing the medication? 4. If you stop the medication, what do you do with the remaining medicine? |
| **Knowledge on danger signs (for both pneumonia and possible serious bacterial infection)** |
| 1. What are the signs for childhood pneumonia that would tell you the child is getting worse or is in danger?   [Mark if the caregiver mentions the following danger signs:]   - Fast breathing - Vomiting - Difficulty eating - Other: ____________________________   What are the signs for possible serious bacterial infection that would tell you the child is getting worse or is in danger?  [Mark if the caregiver mentions the following danger signs:]   - Difficulty in feeding _______ - Vomits everything _______ - Convulsions _______` - Lethargic or unconscious _______   Other: ____________________________   1. What action, if any, would you take if you noticed the following danger signs (*mention all the danger signs in the list*)?   In case of pneumonia  In case of possible serious bacterial infection |
| **Perception of amoxicillin (syrup or DT): general and effectiveness** |
| 1. In your experience how well does amoxicillin (syrup or DT) work to cure pneumonia or possible serious bacterial infection in children? 2. What do you like about amoxicillin (syrup or DT)? 3. What do you dislike about amoxicillin (syrup or DT)? 4. How is it different from the other antibiotics (compared to previous experiences with other antibiotics)? [Caregiver] 5. Does the children like take it? What does it taste like? [Caregiver] |
| **Ease of use of amoxicillin (syrup or DT)** |
| Thinking about each time you gave the amoxicillin (syrup or DT) to your child:   1. What was easy about it? 2. What was difficult about it? |
| **Perception of side effects of amoxicillin (syrup or DT)** |
| 1. How did your child feel while taking the amoxicillin (syrup or DT)? 2. Did you feel that your child reacted to the amoxicillin (syrup or DT) at any time? Please describe what happened. |
| **Amoxicillin formulation preference (syrup vs DT)** |
| 1. *If received syrup:* before today, had you ever heard about amoxicillin DT? From where or whom?   *If received DT*: before you were given amoxicillin DT for your child, had you ever heard about amoxicillin DT? From where or whom?   1. Do you prefer to give your child amoxicillin DT or amoxicillin syrup when treating pneumonia or possible serious bacterial infection? 2. Please describe why you prefer one over the other. *Probe: if one is easier to use than the other, if one works better than the other, etc.* |
| **Factors that influence to use of amoxicillin (syrup or DT)** |
| 1. Where do you get most of your information on how to treat your child when he/she is sick with pneumonia or possible serious bacterial infection? *Probe: healthcare provider, advertisements on radio or tv, what others in the community say.* 2. In the past year, have you seen or heard any information about medication that helps cure pneumonia or possible serious bacterial infection in children? Please describe the names and what the advertisement said. 3. Do you ever follow the recommendations that drug seller give you? |
| Is there anything else you would like to tell me that I forgot to ask? |
| **Documents to ask for review:** |
| Prescription, medicine packet, immunization card or birth certificate (for verifying the age of child) |

Thank the respondent for their assistance.
